# Inotuzumab Ozogamicin as a Bridge to Stem Cell Transplantation in Relapsed Pediatric BCP‐ALL After Tisagenlecleucel: A Case Series

**DOI:** 10.1002/cnr2.70177

**Published:** 2025-03-14

**Authors:** Margo Aertgeerts, Marleen Renard, Anne Uyttebroeck, Nancy Boeckx, Heidi Segers

**Affiliations:** ^1^ Department of Oncology KU Leuven Leuven Belgium; ^2^ Center for Cancer Biology VIB Leuven Belgium; ^3^ Department of Pediatric Hematology and Oncology UZ Leuven Leuven Belgium; ^4^ Department of Laboratory Medicine UZ Leuven Leuven Belgium

**Keywords:** anti‐CD19 CAR T‐cell therapy, inotuzumab ozogamicin, relapsed/refractory BCP‐ALL

## Abstract

**Background:**

CD19‐directed chimeric antigen receptor T‐cell therapy tisagenlecleucel has shown promising results in the treatment of pediatric patients with relapsed/refractory B‐cell precursor acute lymphoblastic leukemia (BCP‐ALL). However, around 50% of patients relapse after tisagenlecleucel. Following multiple relapses, limited treatment options are left, and the prognosis is dismal. We report on four pediatric patients who relapsed after tisagenlecleucel and were treated with inotuzumab ozogamicin (InO).

**Case:**

Four patients with BCP‐ALL received tisagenlecleucel after second relapse (3/4) or refractory disease at first relapse (1/4). Three patients relapsed with CD19^NEG^/CD22^POS^ BCP‐ALL, one with CD19^POS^/CD22^POS^ BCP‐ALL. Following relapse, they received treatment with InO. After the first InO cycle, all achieved complete remission (CR), three without measurable residual disease. After two or three InO cycles, they underwent allogeneic hematopoietic stem cell transplantation (allo‐HSCT). One patient developed an isolated extramedullary relapse (IEM) in both anterior eye chambers six and nine months after allo‐HSCT and received palliative radiotherapy. This patient was in CR at the last follow‐up 25 months later. The other patients were also in CR at the last follow‐up (mean 31.3 months).

**Conclusion:**

InO can be used successfully and safely for the treatment of CD22^POS^ BCP‐ALL relapse after tisagenlecleucel as a bridge to allo‐HSCT in heavily pretreated pediatric patients.

**Abbreviations**
6‐MP: mercaptopurine
Allo‐HSCT: allogeneic hematopoietic stem cell transplantation
Anti‐CD19 CAR T‐cell therapy: CD19‐directed chimeric antigen receptor T‐cell therapy
AR1: average risk group 1
Ara‐C: cytarabine
ATG: anti‐thymocyte globulin
BCA: B‐cell aplasia
BCP‐ALL: B‐cell precursor acute lymphoblastic leukemia
BM: bone marrow
CNS: central nervous system
COG: Children's Oncology Group
CR: complete remission
CSF: cerebrospinal fluid
CSI: craniospinal irradiation
Dexa: dexamethasone
EFS: event‐free survival
EOC: end of consolidation
EOI: end of induction
EORTC: European Organisation for Research and Treatment of Cancer
EOT: end of treatment
GvHD: graft versus host disease
IEM: isolated extramedullary relapse
InO: inotuzumab ozogamicin
IntReALL: international study for treatment of childhood Relapsed ALL
ITCC: Innovative Therapies for Children with Cancer
IT‐MTX: intrathecal methotrexate
LD: lymphodepletion
MFC: multiparameter flow cytometry
MRD: measurable residual disease
MSD: matched sibling donor
MUD: matched unrelated donor
NEG: negative
NGS: next generation sequencing
OS: overall survival
POS: positive
R/R: relapsed/refractory
SOS: sinusoidal obstruction syndrome
SR: standard risk
TBI: total body irradiation
VCR: vincristine
VP‐16: etoposide

## 1 Introduction

Advances in the treatment of B‐cell precursor acute lymphoblastic leukemia (BCP‐ALL), one of the most common pediatric malignancies, have resulted in a 5‐year overall survival (OS) rate of up to 90% [1, 2]. However, relapsed/refractory (R/R) BCP‐ALL in 10%–15% of the children remains one of the greatest therapeutic challenges [3].

One of the immunotherapies in the treatment of R/R BCP‐ALL is CD19‐directed chimeric antigen receptor T‐cell therapy (anti‐CD19 CAR T‐cell therapy). In the breakthrough ELIANA trial, tisagenlecleucel showed an overall remission rate of 81%, a 12‐month event‐free survival (EFS) of 50%, and an overall survival (OS) of 76% in pediatric BCP‐ALL [4]. This led to the approval of tisagenlecleucel for the treatment of R/R BCP‐ALL up to 25 years of age. Since then, real‐world data has shown similar remission rates [5, 6, 7, 8]. However, relapse still occurs in around 40%–50% of patients, mostly within 2 years [6, 7, 9]. Three types of relapse after tisagenlecleucel are described: CD19‐positive (CD19^POS^) relapse (around 50% of cases), CD19‐negative (CD19^NEG^) relapse (around 40%) and relapse with a lineage switch from ALL to AML (around 5%–10%) [6, 7, 9, 10]. In these heavily pretreated children, very few treatment options remain after CAR T‐cell therapy [11].

We present four pediatric patients with R/R BCP‐ALL treated at the University Hospital of Leuven, three presenting with a CD19^NEG^ relapse and one with a CD19^POS^ relapse after treatment with tisagenlecleucel for an earlier relapse. All four patients had CD22‐expressing leukemic blasts and subsequently received inotuzumab ozogamicin (InO). InO is a CD22‐targeted monoclonal antibody linked to calicheamicin, a potent cytotoxic antitumor antibiotic. All patients achieved molecular remission after InO and subsequently underwent allogeneic hematopoietic stem cell transplantation (allo‐HSCT).

## 2 Case Series

### 2.1 Case 1

A 5‐year‐old boy was diagnosed with BCP‐ALL (06/2012) (Table 1, Table S1). He developed a late isolated bone marrow (BM) relapse 3 years and 10 months after the end of primary treatment (EOT) (08/2018). A second isolated BM relapse occurred 30 months after the first relapse with measurable residual disease (MRD) of 1% (03/2021), for which he received tisagenlecleucel. Sixteen months later (07/2022), he developed a combined central nervous system (CNS) and BM (molecular) CD19^POS^/CD22^POS^ relapse (Table 2). He received two cycles of InO and weekly triple intrathecal chemotherapy. After the first InO cycle (three infusions: day 1, day 8, and day 15, total dose 1.8 mg/m^2^), he was in complete remission (CR) without detectable BM MRD. After the second cycle of InO (total dose 1.5 mg/m^2^), he received conditioning with etoposide and total body irradiation (TBI) including craniospinal irradiation (CSI) because of CNS involvement and subsequently underwent allo‐HSCT from a 9/10 matched unrelated donor (MUD) (10/2022). To prevent graft versus host disease (GvHD), pre‐transplant anti‐thymocyte globulin (ATG) and post‐transplant methotrexate (MTX) and cyclosporine were administered. One week after allo‐HSCT, he also developed sinusoidal obstruction syndrome (SOS), but he recovered after 2 weeks of treatment with defibrotide. He was in CR upon last follow‐up 26 months post‐HSCT (12/2024).

**TABLE 1:** Overview of patient history of all cases, showing timing of diagnosis/relapse, subtype of BCP‐ALL (B‐cell precursor acute lymphoblastic leukemia), treatment, and measurable residual disease (MRD) of bone marrow (BM) using PCR (unless otherwise specified).

	Subtype BCP‐ALL	Treatment	BM MRD PCR
Case 1			
Diagnosis (06/2012) Pre‐B‐ALL, B‐other 5‐year‐old boy	CNS1, normal karyotype *CDKN2A* deletion, *ABL1* gain	EORTC 58081 AR1 (NCT01185886) Duration: 28 months (EOT 10/2014)	TP1: < 0.01% TP2: Negative
1st relapse (08/2018) 3 y 10 m after EOT	Late isolated BM relapse	IntReALL SR 2010 (NCT01802814), arm B	TP1: < 0.005% TP2: Negative
26 m after 1st relapse (10/2020)	CNS2 (2% blasts in CSF), BM MRD PCR < 1 × 10^−4^	Weekly IT‐MTX extra	
2nd relapse (03/2021) 30 m after 1st relapse	Isolated BM relapse (MRD PCR 1%, CNS1)	Tisagenlecleucel (lymphodepletion: F + C)	1–6 m after CAR T: Negative
3rd relapse (07/2022) 16 m after anti‐CD19 CAR T‐cell therapy	Combined CNS and BM (molecular) relapse (MRD PCR 0.3%)	2 InO cycles^a^ + weekly triple IT Allo‐HSCT MUD (9/10) (10/2022) Conditioning: TBI + CSI, VP‐16 GvHD prophylaxis: ATG, MTX, cyclosporine	After 1 InO cycle: Negative 3 m–1 y after HSCT: Negative
Case 2			
Diagnosis (08/2013) Pre‐B‐ALL, B‐other 3‐year‐old boy	CNS1, complex pseudodiploid karyotype	EORTC 58081 AR1 Duration: 26 months (EOT 10/2015)	TP1: 0.3% TP2: 0.03%
1st relapse (05/2016) 7 m after EOT	Late isolated BM relapse	IntReALL SR 2010 ➔ IntReALL SR pre‐transplant Allo‐HSCT MSD (10/2016) Conditioning: fludarabine, treosulfan, thiotepa GvHD prophylaxis: cyclosporine	TP1: 0.8% Pre‐HSCT: < 0.001% 1–6 m after HSCT: Negative
2nd relapse (09/2018) 23 m after HSCT	Isolated BM relapse	Ara‐C, 6‐MP, triple IT chemo (MTX/ara‐C/hydrocortisone) as bridge Tisagenlecleucel (lymphodepletion: F + C)	1–12 m after CAR T: Negative
3rd relapse (11/2021) *P2RY8::CRLF2* fusion 3 y after anti‐CD19 CAR T‐cell therapy	Isolated BM relapse Deletion of *ETV6*, *PAR1*, *IKZF1* and *EBF1*, *P2RY8::CRLF2* fusion	3 InO cycles^a^ + VCR, dexa and IT‐MTX Allo‐HSCT MUD (10/10) (03/2022) Conditioning: TBI, VP‐16 GvHD prophylaxis: ATG, MTX, cyclosporine	After 1 InO cycle: 1% After 2 InO cycles: 0.05% After 3 InO cycles: < 0.01% 3 m–1 y after HSCT: Negative
Case 3			
Diagnosis (03/2018) Pre‐B‐ALL, *ETV6::RUNX1* 10‐year‐old girl	CNS1, complex hypodiploid karyotype *TP53* (somatic), *ETV6* and *PAR1* deletion	EORTC 58081 AR1 Duration: 25 months (EOT 04/2020)	TP1: < 0.01% TP2: Negative
1st relapse (10/2020) 6 m after EOT	Late isolated BM relapse	IntReALL SR 2010, ALL R3	TP1: > 1%
Refractory disease (01/2021)		Tisagenlecleucel (lymphodepletion: F + C)	1–6 m after CAR T: Negative
2nd relapse (11/2021) 9 m after anti‐CD19 CAR T‐cell therapy	Isolated (molecular) BM relapse (MRD PCR 3%)	2 InO cycles^a^ + IT‐MTX Allo‐HSCT MUD (9/10) (02/2022) Conditioning: TBI, VP‐16 GvHD prophylaxis: ATG, MTX, cyclosporine	After 1 InO cycle: Negative 3 m–1 y after HSCT: Negative
Case 4			
Diagnosis (01/2019) Common B‐ALL, high hyperdiploid 20 months old girl	CNS2	EORTC 58081 AR1	TP1: Negative TP2: Negative
1st relapse (02/2021) 25 m after diagnosis	Early isolated BM relapse (during maintenance) *IKZF1* and *ETV6* deletion	IntReALL HR, bortezomib arm (NCT03590171)	TP1: Negative
2nd relapse (06/2021) 4 m after 1st relapse	Isolated BM relapse	Ara‐C, 6‐MP, dexa Tisagenlecleucel (lymphodepletion: F + C)	1 m after CAR T, *MRD MFC*: Negative
3rd relapse (11/2021) 4 m after anti‐CD19 CAR T‐cell therapy	Isolated BM relapse	2 InO cycles^a^ + VCR, dexa and IT‐MTX Allo‐HSCT MUD (9/10) (02/2022) Conditioning: TBI, VP‐16 GvHD prophylaxis: ATG, MTX, cyclosporine	After 1 InO cycle: Negative 3–6 m after HSCT: Negative
4th relapse (08/2022 + 11/2022) 6 + 9 m after HSCT	IEM relapse anterior left eye + right eye respectively	Palliative radiotherapy	Negative at time of IEM

Abbreviations: 6‐MP = mercaptopurine, allo‐HSCT = allogeneic hematopoietic stem cell transplantation, AR1 = average risk group 1, ara‐C = cytarabine, ATG = anti‐thymocyte globulin, C = cyclophosphamide, CAR = chimeric antigen receptor, CNS = central nervous system, CSF = cerebrospinal fluid, CSI = craniospinal irradiation, dexa = dexamethasone, EORTC = European Organisation for Research and Treatment of Cancer, EOT = end of treatment, F = fludarabine, GvHD = graft versus host disease, HSCT = hematopoietic stem cell transplantation, InO = inotuzumab ozogamicin, IntReALL = International study for treatment of childhood Relapsed ALL, IT‐MTX = intrathecal methotrexate, m = months, MFC = multiparameter flow cytometry, MSD = matched sibling donor, MUD = matched unrelated donor, SR = standard risk group, TBI = total body irradiation, TP = time point, VCR = vincristine, VP‐16 = etoposide, y = years.

^a^ First cycle of InO was a total dose of 1.8 mg/m^2^ (0.8–0.5—0.5 mg/m^2^ for infusion on day 1—day 8—day 15 respectively). Second and third cycle was a total dose of 1.5 mg/m^2^ (0.5 mg/m^2^ for each infusion on day 1—day 8—day 15).

**TABLE 2:** Overview of relapses after tisagenlecleucel and follow‐up in four patients with relapsed/refractory B‐cell precursor acute lymphoblastic leukemia.

	Case 1	Case 2	Case 3	Case 4
Time to relapse after tisagenlecleucel	16 m	36 m	9 m	4 m
Immunophenotype	CD19^POS^ CD22^POS^	CD19^NEG^ CD22^POS^	CD19^NEG^ CD22^POS^	CD19^NEG^ CD22^POS^
CAR T cells at relapse	Loss (upon relapse)	Detectable (147.6 copies/μg DNA)^a^	Loss (6 m after CAR T)	Detectable (42563.8 copies/μg DNA)^a^
Loss of BCA^b^	Upon relapse	31 m after CAR T	6 m after CAR T	3 m after CAR T
BM MRD PCR after 1st/2nd(/3rd) InO cycle	NEG/NEG	1%/0.05%/< 0.01%	NEG/NEG	NEG/NEG
Follow‐up after allo‐HSCT	26 m CR	33 m CR	35 m CR	IEM after 6 and 9 m 25 m CR after IEM

Abbreviations: allo‐HSCT = allogeneic hematopoietic stem cell transplantation, BCA = B‐cell aplasia, BM = bone marrow, CAR T = chimeric antigen receptor T cells, CR = complete remission, IEM = isolated extramedullary relapse, InO = inotuzumab ozogamicin, m = months, MRD = measurable residual disease, NEG = negative, POS = positive, y = years.

^a^ CAR T cells were detected in the peripheral blood using qPCR of the cytoplasmic domain of the CAR transgene.

^b^ BCA was defined as the absence of CD19^POS^ B cells in the peripheral blood, as detected by immunoflow cytometry.

### 2.2 Case 2

A 3‐year‐old boy was diagnosed with BCP‐ALL (08/2013) (Table 1, Table S1). Seven months after EOT, he developed a late isolated BM relapse (05/2016). Because of poor MRD response after induction treatment according to the IntReALL SR2010 protocol, relapse treatment was consolidated with an allo‐HSCT from a matched sibling donor (MSD) after conditioning with fludarabine, treosulfan, and thiotepa (10/2016). Cyclosporine was given to prevent GvHD. Twenty‐three months after allo‐HSCT (09/2018), he developed a second isolated BM relapse and received tisagenlecleucel. Three years later (11/2021), he developed a third isolated BM relapse, which was CD19^NEG^/CD22^POS^. He still had detectable CAR T cells (Table 2). Genetic/molecular diagnostics, which could be done more extensively, now showed a *P2RY8::CRLF2* fusion and an *IKZF1* deletion. Treatment with InO in combination with conventional chemotherapy was started. After the first InO cycle (total dose 1.8 mg/m^2^), he achieved CR, but BM MRD PCR was still 1%. After the second InO cycle (total dose 1.5 mg/m^2^) BM MRD PCR was 0.05%. After the third InO cycle (total dose 1.5 mg/m^2^), he achieved a molecular remission with MRD PCR < 0.01%, enabling him to continue to the second allo‐HSCT from a 10/10 MUD (03/2022). Conditioning for this allo‐HSCT was performed with TBI and etoposide, and GvHD prophylaxis was achieved with ATG, MTX, and cyclosporine. He was in CR upon the last follow‐up 33 months after allo‐HSCT (12/2024).

### 2.3 Case 3

A 10‐year‐old girl was diagnosed with *ETV6::RUNX1* BCP‐ALL and a somatic *TP53* mutation (03/2018) (Table 1, Table S1). Six months after EOT (10/2020), she developed a late isolated BM relapse and, because of refractory disease, she received tisagenlecleucel. BM MRD was negative until 6 months after tisagenlecleucel, but at that time, she had lost her CAR T cells. BM after 9 months (11/2021) showed a second isolated molecular BM relapse (MRD PCR 0.6%) (Table 2). Two weeks later, MRD had already increased to 3%, and CD19^NEG^/CD22^POS^ blasts were detected. For this second relapse, treatment with InO and intrathecal MTX was started. After the first InO cycle (total dose 1.8 mg/m^2^), she achieved CR, and BM MRD was negative. After the second cycle of InO (total dose 1.5 mg/m^2^), she received conditioning with TBI and etoposide followed by a 9/10 MUD allo‐HSCT (02/2022). She received ATG, MTX, and cyclosporine to prevent GvHD. She was in CR at the last follow‐up 35 months later (01/2025).

### 2.4 Case 4

A girl of 20 months old was diagnosed with high hyperdiploid BCP‐ALL (01/2019) (Table 1, Table S1). During the last month of her maintenance treatment (02/2021), she had an early isolated BM relapse and treatment according to the IntReALL HR protocol was started. After induction treatment, she was in CR with negative MRD. However, because of very severe toxicity with multiple life‐threatening infections, chemotherapy had to be postponed. Four months later (06/2021), she had a second isolated BM relapse. Despite systemic chemotherapy, the disease progressed during the manufacturing time of tisagenlecleucel. Therefore, she received tisagenlecleucel with overt disease (89% blasts in BM).

Three months later, BM showed detectable MRD (PCR < 0.1%). She rapidly progressed with the development of a third isolated CD19^NEG^/CD22^POS^ BM relapse (11/2021) (Table 2). Treatment with InO in combination with chemotherapy was started (Table 1). After the first InO cycle (total dose 1.8 mg/m^2^), she achieved CR, and BM MRD was negative. After the second InO cycle (total dose 1.5 mg/m^2^), she received conditioning with TBI and etoposide and subsequently continued to a 9/10 MUD allo‐HSCT (02/2022). GvHD prophylaxis was achieved with ATG, MTX, and cyclosporine. She remained in CR, and BM MRD was negative 6 months post‐HSCT (08/2022). However, at that time, she developed an isolated extramedullary relapse (IEM) (CD19^NEG^/CD22^POS^) in the left anterior eye chamber, for which she received palliative radiotherapy. Three months later, she had an IEM in the right anterior eye chamber, which was treated similarly. At the latest clinical follow‐up 25 months after the last IEM, she was still in CR (12/2024).

## 3 Discussion

We present four pediatric patients who relapsed with BCP‐ALL after having received tisagenlecleucel for an earlier relapse. Although these patients were heavily pretreated, they all achieved molecular remission after treatment with InO and could proceed to allo‐HSCT as consolidation therapy. Three were in persistent CR at the last follow‐up (mean 31.3 months after allo‐HSCT) and one was in CR 25 months after the last IEM, developed 9 months after allo‐HSCT.

The use of InO in pediatric R/R BCP‐ALL was studied in two Phase II trials, one conducted by the Children's Oncology Group (COG) and one by the Innovative Therapies for Children with Cancer (ITCC) consortium [12, 13]. These studies demonstrated an overall response rate of 58.3% in 48 patients and of 81.5% in 27 patients, respectively, both after the first InO cycle. The COG reported an estimated 2‐year EFS and OS rates of 28.6% and 36.0%, respectively [12]. The ITCC reported a 12‐month EFS and OS rates of 36.7% and 55.1% [13]. In both trials, around 60% of patients proceeded to consolidation therapy (allo‐HSCT and/or CAR T‐cell therapy) after receiving InO [12, 13].

Schultz and colleagues reported about 14 pediatric and young adult patients who, after BCP‐ALL relapse post‐tisagenlecleucel, received InO as salvage treatment [14]. Of these patients, 9/14 achieved CR and 5/14 were non‐responders with progressive disease [14]. Of the patients who achieved CR, 5/9 received subsequent HSCT with a persistent CR in those that were evaluable for follow‐up (3/5) [14]. Our data compare favorably to these other published experiences where InO was used for relapse after tisagenlecleucel but are limited by our sample size. Moreover, one patient only had a molecular BM relapse at the time of InO administration.

A known toxicity of InO is myelosuppression, but in general, InO is tolerated well, with a low incidence of infections [13]. One of the most serious adverse effects of InO is SOS, which is seen more frequently in the case of subsequent allo‐HSCT [13]. Out of pediatric patients receiving allo‐HSCT after InO, 25%–50% develop SOS [12, 13, 15, 16]. In our case series, one patient (1/4) developed SOS after allo‐HSCT but was treated successfully with defibrotide. As part of his conditioning regimen, he received total body irradiation, another risk factor for the development of SOS [17].

InO is promising in the treatment of R/R BCP‐ALL, given its ability to induce remission with relatively lower toxicity in comparison to conventional chemotherapy. New prospective trials are investigating the best implementation of InO in the treatment of pediatric R/R BCP‐ALL (Table 3). Furthermore, in pediatric patients newly diagnosed with BCP‐ALL with a higher risk of relapse, the additional use of InO is now being investigated in frontline therapy with the aim of reducing relapse risk without additional or increased toxicity (Table 3). In adolescents and adult patients of 16 years or older, phase I/II studies are investigating the safety and efficacy of InO after transplantation with the aim of reducing relapse risk and prolonging disease‐free survival (NCT03104491, NCT06427330). The safety of this method was recently shown in a phase I trial [18]. However, pediatric patients are not included in these trials at the moment.

**TABLE 3:** Currently ongoing trials for InO (inotuzumab ozogamicin) in pediatric B‐cell precursor acute lymphoblastic leukemia (BCP‐ALL), either in R/R (relapsed/refractory) patients or in newly diagnosed patients.

	Trial	Goal
InO in R/R BCP‐ALL	Phase I/II study (ITCC‐059, EudraCT 2016‐000227‐71)	Safety and efficacy of InO as single agent or combination with chemotherapy in R/R patients – phase I and IB published [19, 20]
	Phase II study (NCT03913559)	Efficacy of InO in MRD positive (0.1%–4.99%) patients after two prior induction attempts, at relapse, or after allo‐HSCT
	Phase II study (NCT05748171)	Comparison of InO monotherapy to ALLR3 in high‐risk patients at first relapse
	Phase II study (Pedi‐cRIB, NCT05645718)	Efficacy of combination of chemotherapy and compressed rituximab, InO and blinatumomab (cRIB) in relapsed patients
InO for newly diagnosed BCP‐ALL	Phase III study (ALLTogether1, NCT04307576)	Study improved event‐free survival of InO added before maintenance phase in IR (intermediate risk) high group
	Phase III study (COG, NCT03959085)	Study improved disease‐free survival of InO added to post‐induction chemotherapy in high‐risk BCP‐ALL

Abbreviations: COG = Children's Oncology Group, ITCC = Innovative Therapies for Children with Cancer.

## 4 Conclusion

This small cohort of pediatric patients with multiple BCP‐ALL relapses shows that InO can be used successfully and safely as salvage therapy for heavily pretreated children with CD22^POS^ BCP‐ALL relapse after tisagenlecleucel as a bridge to allo‐HSCT. Larger studies with longer follow‐up are needed to further evaluate efficacy and toxicity in this patient group.

## Author Contributions

M.A. and H.S. wrote the manuscript and M.R., A.U. and N.B. critically proofread the manuscript. M.A. performed the literature search and generated all tables.

## Ethics Statement

Free prior written informed consent for publication of this case series was obtained from all patients' parents. With free consent, we mean an unambiguous consent that was obtained from all (parents of) the patients out of their own free will. The obtained consent was documented in the electronic patient file for each participant.

## Consent

Consent for drafting and publication of these case series was obtained from the parents of all patients.

## Conflicts of Interest

The authors declare no conflicts of interest.

## Supporting information


**Table S1.** Complete overview of patient history of all four cases, showing timing of diagnosis/relapse, subtype of B‐cell precursor acute lymphoblastic leukemia (BCP‐ALL) with flow cytometry specifics, central nervous system (CNS) involvement and genetics, presence of chimeric antigen receptor (CAR) T cells and B‐cell aplasia (BCA), treatment protocols, measurable residual disease (MRD) of bone marrow (BM) using PCR or multiparameter flow cytometry (MFC) and follow‐up of patients.

## Data Availability

The data that support the findings of this study are available in the Supporting Information of this article.
